# FCAN–XGBoost: A Novel Hybrid Model for EEG Emotion Recognition

**DOI:** 10.3390/s23125680

**Published:** 2023-06-17

**Authors:** Jing Zong, Xin Xiong, Jianhua Zhou, Ying Ji, Diao Zhou, Qi Zhang

**Affiliations:** 1 Faculty of Information Engineering and Automation, Kunming University of Science and Technology, Kunming 650500, China; zongjing@stu.kust.edu.cn (J.Z.); xiongxin840826@163.com (X.X.); zhoudiao@stu.kust.edu.cn (D.Z.); 20212204324@stu.kust.edu.cn (Q.Z.); 2 Graduate School, Kunming Medical University, Kunming 650500, China; jy970501@163.com

**Keywords:** EEG, emotion recognition, feature fusion, FANet, FCAN–XGBoost

## Abstract

In recent years, artificial intelligence (AI) technology has promoted the development of electroencephalogram (EEG) emotion recognition. However, existing methods often overlook the computational cost of EEG emotion recognition, and there is still room for improvement in the accuracy of EEG emotion recognition. In this study, we propose a novel EEG emotion recognition algorithm called FCAN–XGBoost, which is a fusion of two algorithms, FCAN and XGBoost. The FCAN module is a feature attention network (FANet) that we have proposed for the first time, which processes the differential entropy (*DE*) and power spectral density (*PSD*) features extracted from the four frequency bands of the EEG signal and performs feature fusion and deep feature extraction. Finally, the deep features are fed into the eXtreme Gradient Boosting (XGBoost) algorithm to classify the four emotions. We evaluated the proposed method on the DEAP and DREAMER datasets and achieved a four-category emotion recognition accuracy of 95.26% and 94.05%, respectively. Additionally, our proposed method reduces the computational cost of EEG emotion recognition by at least 75.45% for computation time and 67.51% for memory occupation. The performance of FCAN–XGBoost outperforms the state-of-the-art four-category model and reduces computational costs without losing classification performance compared with other models.

## 1. Introduction

Emotion is a series of reactions that organisms have in response to internal and external stimuli [1]. It can reflect the current psychological and physiological state of human beings and affect daily activities such as cognition, perception, and rational decision-making [2]. Emotion recognition has broad application prospects in fields such as artificial intelligence (AI), intelligent healthcare, remote education, and virtual reality (VR) games [3,4]. Accurately recognizing human emotions is one of the most urgent issues in the brain–computer interface [5].

In early emotion recognition research, researchers mainly used non-physiological signals such as facial expressions [6,7], speech intonation [8], and body movements [9] to recognize emotions and achieved good results. However, the features extracted from these data, such as facial expressions, speech, and body posture, are easy to disguise and are influenced by human subjective factors, making it difficult to reflect the true emotional state. This recognition strategy results in a lack of reliability [10]. In contrast, physiological signals are difficult to disguise and contain more information. Common physiological signals include electroencephalogram (EEG) [11,12,13,14,15,16,17,18,19,20], electromyography (EMG) [21], galvanic skin resistance (GSR) [22], electrocardiogram (ECG) [23], skin temperature (SKT) [24] and pupil diameter [25]. Using physiological signals for emotion recognition results in more reliable results [26]. Among many physiological signals, EEG signals are non-linear, non-stationary, and random signals that record changes in scalp electrical activity. They can reflect human mental state and emotional changes well [27]. More and more researchers are using EEG signals for emotion recognition research and have achieved better results than non-physiological signals such as facial expressions, speech intonation, and body movements [28,29,30,31,32]. While it is true that previous research on emotion recognition using EEG has yielded impressive results, there are still some urgent problems that need to be addressed, such as low recognition accuracy and high computational cost [26]. Given that emotion recognition with a high computational cost has limited practical value, there is a need to develop an EEG-based algorithm for emotion recognition that strikes a balance between high accuracy and low computational requirements.

In this study, we propose an EEG emotion recognition algorithm called FCAN–XGBoost, which is based on multi-frequency band multi-feature fusion and multi-model fusion. FCAN–XGBoost is a hybrid model that combines FCAN for feature processing and eXtreme Gradient Boosting (XGBoost) [33] for feature classification. We conducted four-class experiments on the DEAP [34] and DREAMER [35] public datasets and compared the results with other models. Our experimental findings demonstrate the superior performance of the proposed model in terms of accuracy and computational efficiency. The main contributions of this paper are as follows:

- The differential entropy (*DE*) and power spectral density (*PSD*) features of four EEG frequency bands were extracted, and a parallel feature processing network was proposed to perform further feature extraction and feature fusion on the extracted *DE* features and *PSD* features. We demonstrated the importance of feature fusion in EEG emotion recognition;
- A novel feature attention network (FANet) was proposed to assign different weights to features of varying importance levels. This was performed to enhance the expression ability of features, and it was proven to improve the accuracy of EEG emotion recognition;
- A novel FCAN–XGBoost hybrid EEG emotion classification network was proposed. This network was shown to consume fewer computing resources, while still possessing strong accuracy and robustness in EEG emotion recognition;
- Extensive four-class classification experiments were conducted on the DEAP and DREAMER public datasets. The experimental results demonstrate dthat the FCAN–XGBoost hybrid model is superior to existing models and significantly reduces the computational cost of emotion recognition.

## 2. Related Work

The selection of feature extraction methods and classification algorithms plays a pivotal role in the outcome of the EEG emotion recognition task [15]. Over the past few years, researchers have conducted extensive investigations into the selection of appropriate EEG feature extraction methods and classification algorithms [36,37,38,39,40,41,42,43,44,45,46,47,48,49,50,51]. Such efforts have yielded significant advancements in this field.

### 2.1. Different Features for EEG Emotion Recognition

EEG can reflect the electrophysiological activity of brain nerve cells in the cerebral cortex or scalp surface [27]. Human emotion changes and brain nerve activity are closely related, and EEG records the state changes of brain nerve cells during emotion changes in real-time; this signal is very realistic and has a high temporal resolution. Therefore, the results of emotion recognition by EEG are more accurate and reliable [15]. Typically, time-domain features, frequency-domain features, time–frequency features, nonlinear features, or a combination of these features are extracted from EEG signals for this purpose [14,15]. Mehmood et al. [16] employed the Hjorth parameter to extract EEG signal features and utilized random forests for the binary classification of emotions. Their study encompassed binary classification experiments on DEAP, SEED-IV, DREAMER, SELEMO, and ASCERTAIN datasets, with corresponding accuracy rates of 69%, 76%, 85%, 59%, and 87%. Tripathi et al. [17] extracted nine features, comprising the mean, median, maximum, minimum, standard deviation, variance, value range, skewness, and kurtosis, from the DEAP EEG signal. They employed deep neural networks (DNN) and convolutional neural networks (CNN) for two classifications and attained superior results. Gao et al. [18] extracted fuzzy entropy (FE) and *PSD* from high-frequency EEG signals and applied multi-order detrended fluctuation analysis (MODFA) to classify emotions. Their study achieved an accuracy rate of 76.39% in the three-category task. Bai et al. [19] extracted *DE* features from EEG signals of the DEAP dataset and utilized a residual network with deep convolution and point convolution for binary classification, with an accuracy rate of 88.75%. Fraiwan et al. [3] used multiscale entropy (MSE) to extract features from EEG, principal component analysis (PCA) for feature dimension reduction, and, finally, artificial neural networks (ANNs) to predict the enjoyment of museum pieces, obtaining a high 98.0% accuracy.

### 2.2. Fusion Features for EEG Emotion Recognition

Extracting multiple features of EEG and fusing them with different fusion strategies often results in better emotion recognition than single features [20]. Multi-band feature fusion has particularly demonstrated effectiveness in enhancing the accuracy of emotion recognition [28]. An et al. [29] proposed an EEG emotion recognition algorithm based on 3D feature fusion and convolutional autoencoder (CAE), which extracted *DE* from different frequency bands and fused them into 3D features. Using CAE for emotion classification, the recognition accuracy rates of valence and arousal dimensions on the DEAP dataset were 89.49% and 90.76%, respectively. Gao et al. [30] developed a method of fusing power spectrum and wavelet energy entropy to classify three emotions (neutral, happy, and sad) using support vector machine (SVM) and relational vector machine (RVM). The experimental results showed that the fusion of two features was superior to a single feature. Zhang et al. [31] proposed a multi-band feature fusion method GC–F-GCN based on Granger causality (GC) and graph convolutional neural network (GCN) for emotional recognition of EEG signals. The GC–F-GCN method demonstrated superior recognition performance than the state-of-the-art GCN method in the binary classification task, achieving average accuracies of 97.91%, 98.46%, and 98.15% for arousal, valence, and arousal–valence classification, respectively. Parui et al. [32] extracted various features, including frequency domain features, wavelet domain features, and Hjorth parameters, and used the XGBoost algorithm to perform binary tasks on the DEAP dataset. The accuracy rates of valence and arousal reached 75.97% and 74.206%, respectively. These findings suggest that the use of multiple features and their fusion through appropriate strategies can significantly enhance the recognition accuracy of emotions using EEG signals.

### 2.3. Hybrid Model for EEG Emotion Recognition

In addition to the technique of feature fusion, the application of hybrid models has been proven to be effective in improving the accuracy of emotion recognition [36,37,38]. Various studies have explored this approach and achieved promising results. For example, Chen et al. [39] proposed a cascaded and parallel hybrid convolutional recurrent neural network (CRNN) for binary classification of EEG signals using spatiotemporal EEG features extracted from the *PSD* of the signals. The proposed hybrid networks achieved classification accuracies of over 93% on the DEAP dataset. Similarly, Yang et al. [40] developed a hybrid neural network that combined a CNN and a recurrent neural network (RNN) to classify emotions in EEG sequences. They converted chain-like EEG sequences into 2D frame sequences to capture the channel-to-channel correlation between physically adjacent EEG signals, achieving an average accuracy of 90.80% and 91.03% for potency and arousal classification, respectively, on the DEAP dataset. Furthermore, Wei et al. [42] proposed a transformer capsule network (TCNet) that consisted of an EEG Transformer module for feature extraction and an emotion capsule module for feature refinement and classification of emotional states. On the DEAP dataset, their proposed TCNet achieved average accuracies of 98.76%, 98.81%, and 98.82% for binary classification of valence, arousal, and dominance dimensions, respectively. These studies demonstrate the potential of hybrid models in enhancing the performance of emotion recognition.

### 2.4. Multi-Category EEG Emotion Recognition

Compared to the research focusing solely on binary emotions, multi-classification research on emotions has promising prospects [42,43,44,45]. Hu et al. [46] introduced a hybrid model comprised of a CNN, a bidirectional long short-term memory network (BiLSTM), and a multi-head self-attention mechanism (MHSA) which transforms EEG signals into temporal frequency maps for emotion classification. The model achieved an accuracy rate of 89.33% for the four-category task using the DEAP dataset. Similarly, Zhao et al. [47] proposed a 3D convolutional neural network model to automatically extract spatiotemporal features in EEG signals, achieving an accuracy rate of 93.53% for the four-category task on the DEAP dataset. Singh et al. [48] utilized SVM to classify emotions by extracting the different features of EEG average event-related potentials (ERPs) and average ERPs, achieving accuracy rates of 75% and 76.8%, respectively, for the four-classification tasks on the DEAP dataset. Gao et al. [49] proposed a new strategy for EEG emotion recognition that utilized Riemannian geometry. Wavelet packets were used to extract the time–frequency features of EEG signals to construct a matrix for emotion recognition, achieving an accuracy rate of 86.71% for the four-category task on the DEAP dataset.

In conclusion, there remains ample opportunity to enhance the precision of EEG-based emotion recognition. To this end, we present a novel hybrid model, the FCAN–XGBoost, aimed at improving the accuracy of four-category EEG emotion recognition while minimizing computational costs. Unlike previous work in this area, we propose the FCAN–XGBoost model to achieve this goal, which is a novel hybrid model for four-category EEG emotion recognition using EEG fusion features.

## 3. Materials and Methods

### 3.1. Model Overview

**Figure 1:**
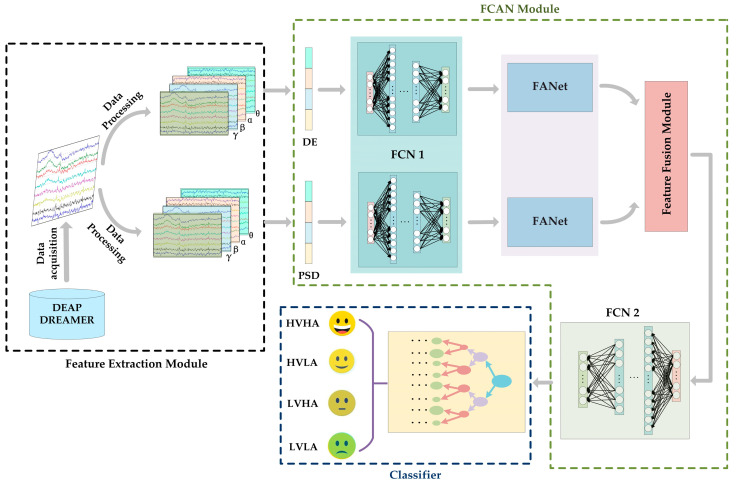
FCAN–XGBoost model framework diagram. Our model consists of three parts: feature extraction module, FCAN module, and classifier. Among them, FCAN module consists of FCN1, FANet, feature fusion module, and FCN2.

Figure 1 shows the overall framework and flow of the proposed new model FCAN–XGBoost is comprised of three modules, namely, the feature extraction module, the FCAN module, and classifier. The feature extraction module is tasked with extracting pertinent features from EEG signals across various frequency bands, whereas the FCAN module is responsible for the comprehensive processing and fusion of features. The function of classifier, on the other hand, is to facilitate the classification of emotion. A detailed account of the framework and process of the proposed model is provided in subsequent sections.

### 3.2. EEG Datasets

DEAP and DREAMER datasets have been widely used in EEG-based emotion recognition research [16,42,43,44,45]. Therefore, we validated our proposed FCAN–XGBoost algorithm on the DEAP and DREAMER datasets.

DEAP is a multimodal dataset consisting of 32 participants watching 40 one-minute music videos. The dataset consists of a range of physiological signals that include galvanic skin response, EEG, EMG, electrooculogram (EOG), skin temperature, blood volume pressure, and respiration rate. The EEG signals were recorded from 32 electrodes in accordance with the international 10–20 system at a sampling rate of 512 Hz. Additionally, each participant used the self-assessment manikin (SAM) to rate their emotional arousal, valence, liking, and dominance for every trial. The participants provided numerical scores between 1 to 9 to indicate their emotional states.

DREAMER is a multimodal dataset that encompasses 23 participants, each of whom underwent 18 distinct trials. EEG signals were acquired using a wearable, low-cost EEG acquisition device comprising 14 EEG channels, which were sampled at a frequency of 128 Hz. Similar to the DEAP dataset, participants’ emotional states were evaluated via a continuous emotion model, and each participant was asked to rate their emotions on three dimensions (arousal, potency, and dominance) using the SAM scale, ranging from 1 to 5, for each trial.

### 3.3. Feature Extraction Module

The function of the feature extraction module in the emotion recognition model is primarily to extract EEG signal features and engage in the data processing. Previous studies have demonstrated the efficacy of *DE* and *PSD* features in EEG-based emotion recognition [29,39]. The *PSD* of EEG signals reflects the distribution of EEG signal power in different frequency bands [52]. The *DE* [53] of an EEG signal is an extension of Shannon entropy on continuous variables. For a specific length of EEG signal that approximately follows a Gaussian distribution, its *DE* is equal to the logarithm of its energy spectrum in a specific frequency band. Notably, *PSD* and *DE* features are the most widely used features in the field of EEG emotion recognition. As a result, in this study, we extracted *DE* and *PSD* features of EEG signals from the DEAP and DREAMER public datasets for the subsequent emotion recognition process.

Equation (1) delineates the equation to calculate the *DE* of an EEG signal segment of length [a,b] that closely conforms to a gaussian distribution $N(\mu ,\sigma^{2})$:

(1) $$DE=-\int_{a}^{b}\frac{1}{\sqrt{2\pi \sigma_{i}^{2}}}e^{-\frac{{\left(x-\mu \right)}^{2}}{2\sigma_{i}^{2}}}log(\int_{a}^{b}\frac{1}{\sqrt{2\pi \sigma_{i}^{2}}}e^{-\frac{{\left(x-\mu \right)}^{2}}{2\sigma_{i}^{2}}})dx=\frac{1}{2}log(2\pi e\sigma_{i}^{2})$$

Assuming an EEG signal of length M, denoted as x(t), if we consider the value of t to be 0~M−1, the *PSD* of the signal can be determined using Equation (2):

(2) $$P\left(\omega_{k}\right)=\overset{M-1}{\underset{t=-\left(M-1\right)}{\sum }}\gamma \left(t\right)e^{-j\omega_{k}t}$$

where the variable $P(\omega_{k})$ is the power spectral density; γ(t) denotes the autocorrelation function of x(t); k=−(M−1),−(M−2),⋅⋅⋅,0,1,⋅⋅⋅,M−1; $\omega_{k}$ is angular frequency; and t denotes time.

### 3.4. FCAN Module

The feature processing module encompasses four individual sub-modules, namely FCN1, FANet, feature fusion model, and FCN2.

#### 3.4.1. FCN1

The fundamental architecture of FCN1 is a multi-layered fully connected neural network (FCN) [54] comprising an input layer, a hidden layer, and an output layer. Assuming that the input of the l-th neuron in the i-th layer is $x_{i}^{l}$ and the output is $y_{i}^{l}$ gives the following:

(3) $$y_{i}^{l}=\sigma \left(\overset{n}{\underset{j=1}{\sum }}w_{ij}^{l}x_{j}^{l-1}+b_{i}^{l}\right)$$

where n is the number of neurons in the l−1-th layer; $w_{ij}^{l}$ is the weight between the l-th neuron in the i-th layer and the l−1-th neuron in the j-th layer; $b_{i}^{l}$ is the bias of the l-th neuron in the i-th layer; and σ is the ReLU [54] activation function.

The training procedure of FCN primarily consists of two fundamental steps: forward propagation and backpropagation [55]. During forward propagation, the network computes the output values based on the input data. In contrast, backpropagation aims to minimize the error between the predicted and actual output by adjusting the network’s weight and bias parameters. This iterative process involves computing the gradient of the loss function with respect to the network’s parameters and updating them, accordingly, to minimize the objective function.

Suppose the training data set is $D=\{(x_{1},y_{1}),(x_{2},y_{2}),\ldots ,(x_{m},y_{m})\}$, where $x_{i}$ is the input data and $y_{i}$ is the corresponding label. The objective of FCN is to minimize the difference between the output value generated by the network and the actual value. This can be achieved by minimizing the loss function L, which is defined as follows:

(4) $$L=\frac{1}{m}\overset{m}{\underset{i=1}{\sum }}l\left(y_{i},{\hat{y}}_{i}\right)$$

where L denotes the training loss; $l\left(y_{i},{\hat{y}}_{i}\right)$ denotes the loss per prediction; and $y_{i}$ and ${\hat{y}}_{i}$ are the actual and predicted values, respectively.

Fully connected neural networks have high flexibility, high adaptivity, high interpretability, high scalability, and high robustness in processing EEG data and can flexibly adjust the model structure by regularizing and increasing the number of fully connected layers or nodes to adapt to more complex EEG data and tasks. Thus, two different fully connected neural networks, FCN1 and FCN2, were used in this study to process the features.

**Figure 2:**
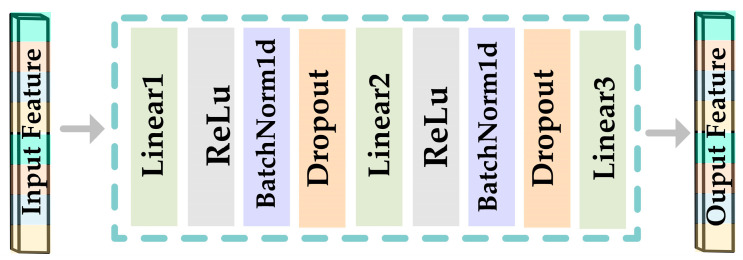
Framework diagram of FCN1.

The FCN1 network consists of two fully connected networks with the same structure, which are mainly responsible for the initial processing of *DE* features and *PSD* features; its structure is shown in Figure 2, and the detailed parameters are shown in Table 1.

The FCN1 module, as illustrated in Figure 1, consists of two fully connected neural networks with identical structures running in parallel. They are responsible for separately processing the *DE* and *PSD* features extracted from the feature extraction module. The input shape of the network that processes *DE* features in FCN1 is (128, ), while the input shape of the network responsible for processing *PSD* features is (56, ). Both *DE* and *PSD* features processed by FCN1 produce an output shape of (128, ). The preliminary processing of *DE* and *PSD* features by the FCN1 module can yield a more profound representation of these features.

#### 3.4.2. FANet

**Figure 3:**
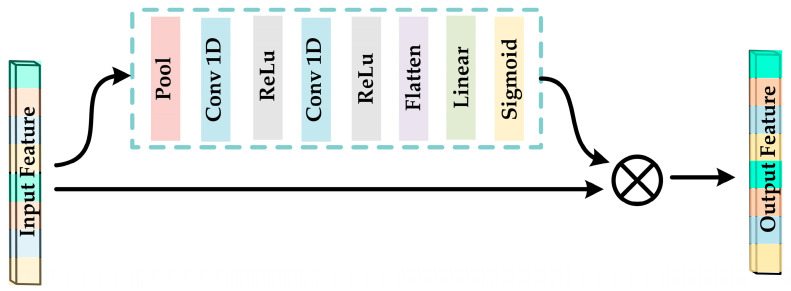
Framework diagram of FANet.

Motivated by the channel attention mechanism proposed in prior works [56], we aimed to enhance the descriptive potential of features by introducing the FANet into the feature processing module after FCN1. The architecture of the FANet is presented in Figure 3, while the corresponding layer-wise parameter configurations are provided in Table 2.

By imparting distinct weights to the processed *DE* and *PSD* features, the FANet amplifies the salient features while attenuating the less significant ones. This leads to an improved recognition ability in the emotion classifier. FANet consists of two parallel submodules with the same structure, one for processing *DE* features from FCN1 and the other for processing *PSD* features from FCN1. Both submodules output a shape of (128, ).

#### 3.4.3. FCN2

**Figure 4:**
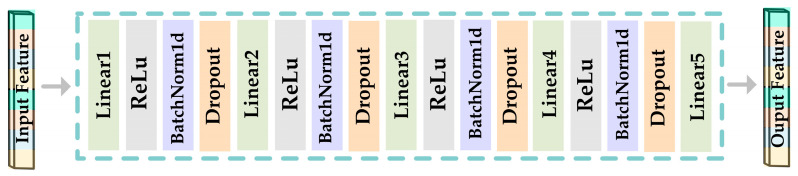
The framework diagram of FCN2.

FCN2 comprises a multi-layer FCN. The fusion of the two features results in a doubling of their dimensions. However, high-dimensional EEG features are typically unsuitable for emotion classification. As a result, prior to feeding to the classifier, dimensionality reduction is necessary. In this research, the aim was to attain both the effect of feature dimensionality reduction and the acquisition of emotionally expressive features. Therefore, conventional algorithms such as stacked autoencoder (SAE) [5] or principal component analysis (PCA) [3] were not employed to reduce the dimensionality of the fusion features. Instead, an FCN2 network with an output dimension of four was introduced after the feature fusion module to obtain low-dimensional features with a more pronounced emotional expression capacity. Figure 4 depicts the structure of the FCN2 network, while Table 3 shows the detailed parameters.

#### 3.4.4. Feature Fusion Module

The most frequently used feature fusion approaches in deep learning-based algorithms are feature vector addition, multiplication, and concatenation [30]. This research investigates the influence of different feature fusion strategies on the efficacy of emotion recognition through experimental analysis. Following a meticulous comparison of the results, the method of feature concatenation was ultimately selected for implementation, which can be defined using Equations (5)–(7):

(5) $$X_{DE}=\left[d_{1},d_{2},d_{3},\ldots ,d_{128}\right]$$

(6) $$X_{PSD}=\left[p_{1},p_{2},p_{3},\ldots ,p_{128}\right]$$

(7) $$X_{con}=\left[X_{DE},X_{PSD}]=[d_{1},d_{2},d_{3},\ldots ,d_{128},p_{1},p_{2},p_{3},\ldots ,p_{128}\right]$$

where the *DE* features are denoted by $X_{DE}$ and the *PSD* features are denoted by $X_{PSD}$. The final set of features obtained after the fusion of these individual feature sets is denoted by $X_{con}$.

As described in Equations (5)–(7), the feature fusion module is used to fuse the *DE* and *PSD* features from FANet, and the fusion strategy used by the feature fusion module is concatenation. The *DE* and *PSD* features of shape (128, ) from the FANet module are processed by the feature fusion module and fused into a new feature vector of shape (256, ).

### 3.5. Classifier

The classification algorithm we chose was XGBoost. XGBoost is an ensemble learning algorithm based on decision trees and is an enhanced version of the gradient boosting algorithm. The algorithm’s fundamental principle is to construct a robust classifier by combining multiple weak classifiers. At each iteration, XGBoost refines the weights of each weak classifier based on the current model’s performance, thereby enhancing the subsequent iteration’s capacity to fit the data optimally. The specific mathematical expression of the XGBoost algorithm is presented below:

(8) $${\hat{y}}_{i}^{\left(t\right)}={\hat{y}}_{i}^{\left(t-1\right)}+f_{t}(x_{i})$$

(9) $$Obj=\overset{n}{\underset{i=1}{\sum }}l\left(y_{i},{\hat{y}}_{i}{}^{\left(t-1\right)}\right)+f_{t}\left(x_{i}\right)+\Omega \left(f_{t}\right)$$

where ${\hat{y}}_{i}{}^{\left(t\right)}$ denotes the predicted value of the *t*-th iteration, ${\hat{y}}_{i}{}^{\left(t-1\right)}$ denotes the predicted value of the *t* − 1 iteration, and $f_{t}(x_{i})$ denotes the prediction of the *t*-th decision tree, which is integrated into the model. The objective function, Obj, is incorporated into the model to assess the degree of fitting of the model. Additionally, a regularization term, $\Omega \left(f_{t}\right)$, is included to regulate the complexity of the model. Lastly, $f_{t}$ denotes the *t*-th decision tree used in the model.

XGBoost has significantly improved modeling efficiency compared to the general gradient boosting decision tree (GBDT) [57], surpassing the random forest (RF) [58] model by more than double and achieving ten times faster performance than GBDT. Consequently, we selected XGBoost as the classifier to enhance the efficiency of emotion recognition.

## 4. Experiments

### 4.1. Data Processing

In this study, we decomposed the EEG signals into four frequency bands: theta (4–8 Hz), alpha (8–14 Hz), beta (14–31 Hz), and gamma (31–45 Hz). Theta waves have a frequency range of 4–8 Hz and are present in the brain’s frontal lobe when individuals experience mental relaxation or light sleep. Alpha waves have a frequency range of 8–14 Hz and typically appear in the brain’s occipital lobe when individuals are calm and awake. Beta waves have a frequency range of 14–31 Hz and usually appear in the brain’s frontal lobe when individuals are mentally tense or emotionally excited. Gamma waves have a frequency range of 31–45 Hz and typically occur when individuals are focused or alert [12,59].

The emotions were categorized into four categories: high valence high arousal (HVHA), high valence low arousal (HVLA) [43], low valence high arousal (LVHA), and low valence low arousal (LVLA). HVHA corresponds to the excitement, which occurs when the participant is in a high valence and high arousal state during the experiment. HVLA represents calmness or relaxation, which occurs when the participant is in a high valence and low arousal state during the experiment. LVHA corresponds to anger or depression, which occurs when the participant is in a low valence and high arousal state during the experiment. LVLA represents sadness and dejection, which occurs when the participant is in a low valence and low arousal state during the experiment.

#### 4.1.1. DEAP Dataset Processing

When processing EEG data from the DEAP dataset, a cut-off point of five was utilized, whereby labels below five were assigned a value of zero, and those above five were given a value of one. Four distinct emotions were classified, including HVHA, HVLA, LVHA, and LVLA, which were labeled as 0, 1, 2, and 3, respectively. The EEG signals used in the experiment underwent downsampling to 128 Hz, while EOG artifacts were removed, and a band-pass filter ranging from 4 Hz to 45 Hz was applied for filtering. The EEG signal of each participant was decomposed into four frequency bands: theta, alpha, beta, and gamma, after which the EEG signal was intercepted with a 2 s [60] non-overlapping time window.

In the DEAP dataset, each trial spanned 63 s, comprising a 3 s baseline time before the start of the experiment and a 60 s stimulus time. The first 3 s of the EEG signal were utilized as the baseline signal, from which the *DE* and *PSD* features for the first 3 s and the second 60 s were extracted, respectively. The final features are obtained by subtracting the *DE* and *PSD* feature values of the baseline time from the *DE* and *PSD* feature values of the stimuli time. Consequently, four *DE* and *PSD* feature values for each channel could be extracted from each time window for each subject. Ultimately, a *DE* feature vector of length 128 and a *PSD* feature vector of length 128 were extracted from all 32 channels, resulting in 38,400 *DE* feature samples and 38,400 *PSD* feature samples for the 32 subjects in the DEAP dataset.

#### 4.1.2. DREAMER Dataset Processing

When processing EEG data from the DEAP dataset, we used 2.5 as the cut-off point; labels below 2.5 were assigned a value of 0, and labels above 2.5 were assigned a value of 1. When processing EEG data from the DREAMER dataset, a cut-off point of 2.5 was employed, with labels below 2.5 assigned a value of 0 and those above 2.5 assigned a value of 1. Four distinct emotions were classified in this experiment, namely, HVHA, HVLA, LVHA, and LVLA, which were labeled as 0, 1, 2, and 3, respectively. Each trial in the DREAMER dataset consisted of 61 s of baseline time and 65 s to 393 s of stimuli time. We used the baseline EEG signal of each trial as the base signal, extracted the *DE* and *PSD* features of the baseline and stimulus signals, and subtracted the simulated *DE* and *PSD* features from the baseline *DE* and *PSD* features as our final features. Finally, a *DE* feature vector of length 56 and a *PSD* feature vector of length 56 were extracted from all 14 channels. With 23 subjects in the DREAMER dataset, a total of 42,803 *DE* feature samples and 42,803 *PSD* feature samples were obtained.

### 4.2. Baseline Model

To ascertain the efficacy of the proposed classification model, a comparative analysis was conducted between the classification performance of the proposed FCAN–XGBoost model and two other models, namely FCAN–SVM and FCAN–LSTM. To ensure an equitable evaluation, the same data processing techniques, and experimental setup were employed for all baseline models.

#### 4.2.1. FCAN–SVM

In previous studies on EEG emotion recognition, SVM [61] has been widely utilized as a classification model with promising results [18,29,47]. In this study, we introduce a novel approach by fusion the FCAN module with the SVM algorithm to propose the FCAN–SVM algorithm. The FCAN–SVM algorithm was subsequently utilized as one of the baseline models for the experiment.

#### 4.2.2. FCAN–LSTM

Long short-term memory (LSTM) [62] is a variant of the recurrent neural network (RNN) [63] architecture. It was first introduced by Hochreiter and Schmidhuber in 1997 and has undergone various optimizations and improvements by researchers over the years. LSTM is particularly adept at learning long-term dependencies, making it a suitable algorithm for processing and predicting time series data. Many researchers have utilized the LSTM in their studies, including EEG emotion recognition studies [39,46]. In this study, we present the FCAN–LSTM model, which fuses the LSTM algorithm with the FCAN module. The LSTM component of our model comprises two LSTM layers and a connection layer. We also employ this architecture as a benchmark model for our experiments.

### 4.3. Performance Evaluation Metrics

To evaluate the classification performance of the model comprehensively and objectively, we treated the four classification tasks as separate binary classification problems and used the following evaluation metrics: accuracy, precision, recall, and F1-score. Accuracy represents the proportion of samples correctly predicted by the model. Precision refers to the fraction of correctly predicted positive samples among all samples that the model predicts as positive, while recall denotes the fraction of correctly predicted positive samples among all actual positive samples. The F1-score is a performance metric that takes into account both precision and recall in its calculation. These four metrics are defined below:

(10) $$\mathrm{Accuracy}=\frac{TP+TN}{TP+FP+TN+FN}$$

(11) $$\mathrm{Pr}\mathrm{ecision}=\frac{TP}{TP+FP}$$

(12) Recall=TPTP+FN

(13) F1−score=2×Precision×RecallPrecision+Recall

where TP, FN, TN, and FP denote true positives, false negatives, true negatives, and false positives, respectively.

In addition, to assess the internal consistency and reliability of the measures or scales used in the study, we evaluated the results of our experiments using Cronbach’s alpha, a statistical indicator of the internal consistency of a measurement instrument. Typically, it takes on a value between 0 and 1, with larger values representing the higher reliability of the measurement instrument.

## 5. Results and Discussion

We trained the model on an NVIDIA GTX 1080ti GPU. The learning rates f for FCAN and XGBoost were set to 0.001 and 0.25, respectively, and a dynamic learning rate adjustment mechanism was used during the model training. The optimization function was set to Adam optimization. The loss function was set to cross-entropy. In our experiments, we divided the data into training and test sets in the ratio of 8:2.

### 5.1. Ablation Experiments

To objectively verify the classification effect of our model, we conducted three kinds of ablation experiments on the DEAP and the DREAMER. The first experiment was to explore the influence of different feature fusion methods on the accuracy of emotion recognition; the second was to verify the effect of the FANet module on emotion recognition; the third was to explore the influence of the position of the fully connected layer in the FCN2, where the XGBoost algorithm is located in the classification module, on the experimental results.

#### 5.1.1. Feature Fusion Ablation Experiments

The experiments were performed on two data sets for emotion classification using only *DE* features, emotion classification using only *PSD* features, and emotion classification using the addition, multiplication, and concatenation of *DE* and *PSD* features. The results of the experiments are shown in Table 4 and Table 5.

In Table 4 and Table 5, *DE* represents emotion recognition using *DE* features only, *PSD* represents emotion recognition using *PSD* features only, $X_{add}$ represents emotion classification by adding *DE* and *PSD* features, $X_{mult}$ represents emotion classification by multiplying *DE* and *PSD* features, and $X_{con}$ represents emotion classification by concatenating *DE* and *PSD* features.

Table 4 and Table 5 show that the accuracy of emotion classification using only *DE* and *PSD* features was lower than the classification achieved by fusing the two features. Furthermore, compared to emotion classification using the multiplication of the two features, the addition of *DE* and *PSD* features did not lead to higher accuracy. In particular, the best results were obtained by concatenating *DE* and *PSD* features for emotion classification. DEAP and DREAMER datasets achieved the highest accuracies of 95.26% and 94.05%, respectively. These results demonstrate that the concatenation of *DE* and *PSD* features can significantly improve the accuracy of EEG-based emotion recognition. The Cronbach’s alpha values in both Table 4 and Table 5 were 0.99, thus showing the high internal consistency and reliability of the measures used in this study.

#### 5.1.2. FANet Module Ablation Experiments

Experimental results are shown in Table 6 and Table 7. The impact on emotion classification was investigated by including and excluding FANet modules in feature processing modules and by placing FANet modules in different positions.

In Table 6 and Table 7, FCAN–XGBoost represents the model with the FANet module, FCN–XGBoost represents the model without the FANet module, and AF represents the experimental results with the FANet module placed after the feature fusion module.

Table 6 and Table 7 show that, for the DEAP and DREAMER datasets, the emotion recognition accuracy with the FANet module improved by 0.48 and 1.35 percentage points, respectively, compared to those without the FANet module. This indicates that the inclusion of the FANet module in the feature processing module helped to improve the classification performance of the model emotion. The Cronbach’s alpha values in Table 6 and Table 7 are 0.96 and 0.99, respectively, indicating high measurement consistency and reliability.

#### 5.1.3. Impact of XGBoost Algorithm at Different Positions in FCN2

As seen in Section 3.4.3, there are five fully connected layers in FCN2 in FCAN–XGBoost. We fed the outputs of the different fully connected layers in FCN2 into the XGBoost classifier for an emotion classification experiment, and the experimental results are shown in Table 8.

Table 8 shows the experimental results, where various combinations of fused features and fully connected layers were evaluated for emotion classification. Specifically, No_FC represents when the fused features were directly fed into the XGBoost classifier without passing through the FCN2 network. In contrast, IFC1, IFC2, IFC3, IFC4, and IFC5 refer to the scenarios where the outputs of the first, second, third, fourth, and last fully connected layers in FCN2 were used for emotion classification, respectively. The Cronbach’s alpha value of 0.97 in Table 8 also demonstrates good consistency and reliability of the measurements. The FCN2 network aims to reduce the dimensionality of the fused features and enhance their emotional expressiveness, leading to improved performance in emotion classification. Our experimental results validate the effectiveness of the proposed FCAN–XGBoost algorithm.

### 5.2. Comparative Experiments

We comparef the proposed emotion classification model with two baseline models and the state-of-the-art emotion classification models.

Table 9 and Table 10 provide a comprehensive account of the relative time and memory consumption by each model employed for the task of emotion recognition. Specifically, the metric of “Time” signifies the duration taken by each model to perform the task of emotion recognition on the test set, whereas “Memory” represents the extent of memory space occupied by each model in executing the task of emotion recognition on the test set. Notably, our proposed model outperformed the two baseline models on the DEAP and DREAMER datasets, in terms of achieving higher accuracy in emotion recognition while requiring fewer computational resources, as evidenced by its relatively lesser memory usage and shorter computation time.

**Figure 5:**
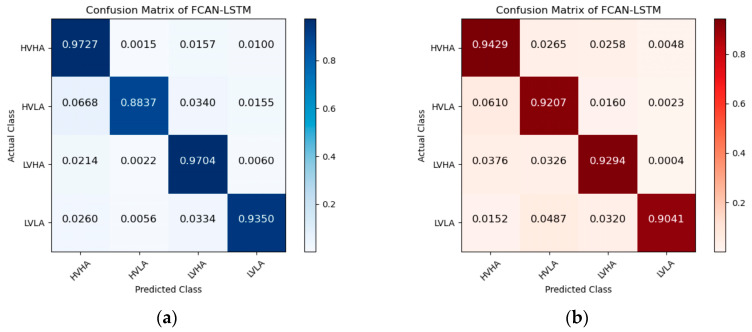
Confusion matrices of FCAN–LSTM in the experiments on DEAP and DREAMER: (**a**) the confusion matrix of the experiment conducted on the DEAP dataset; (**b**) the confusion matrix of the experiment conducted on the DREAMER dataset.

**Figure 6:**
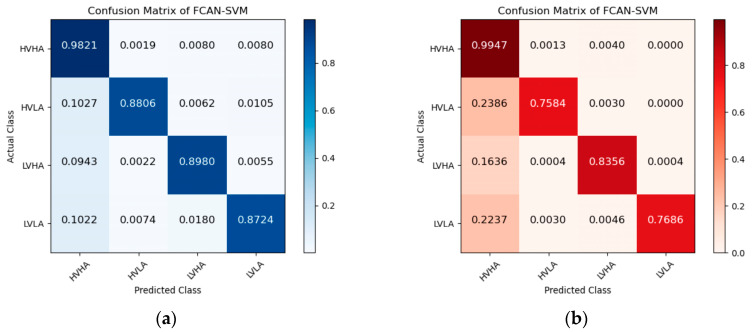
Confusion matrices of FCAN–SVM in the experiments on DEAP and DREAMER: (**a**) the confusion matrix of the experiment conducted on the DEAP dataset; (**b**) the confusion matrix of the experiment conducted on the DREAMER dataset.

**Figure 7:**
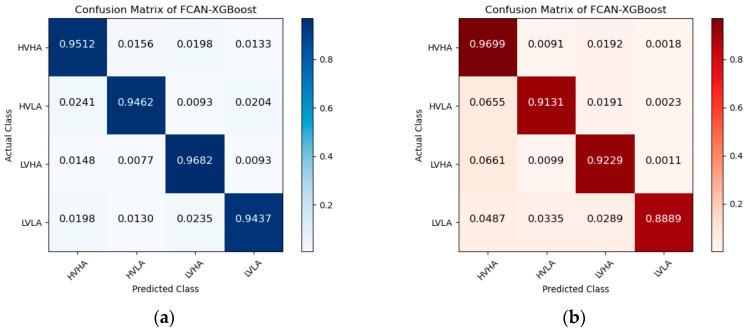
Confusion matrices of FCAN–XGBoost in the experiments on DEAP and DREAMER: (**a**) the confusion matrix of the experiment conducted on the DEAP dataset; (**b**) the confusion matrix of the experiment conducted on the DREAMER dataset.

Figure 5, Figure 6 and Figure 7 are the confusion matrices of FCAN–LSTM, FCAN–SVM, and FCAN–XGBoost for four-category emotion recognition on the DEAP and DREAMER datasets. The findings indicate that the proposed FCAN-based emotion recognition model exhibits significantly superior classification performance than the two baseline models.

Furthermore, we compared the emotion recognition model of the proposed model with the state-of-the-art models. The results are shown in Table 11.

Table 11 displays the comparative analysis of our model with other existing models [16,42,43,44,45,46,47,48,50,51], and it illustrates that our proposed model exhibits exceptional performance in recognizing emotions. We conducted the emotion four-category task on two widely used datasets, namely, DEAP and DREAMER, and the attained accuracy rates were 95.26% and 94.05%, respectively. These results demonstrate the efficacy of our FCAN–XGBoost emotion classification model in recognizing four-category emotions.

## 6. Conclusions

This paper proposes a novel emotion recognition model named FCAN–XGBoost. A feature fusion strategy was employed to obtain fusion features. Motivated by the channel attention mechanism, we first proposed FANet to assign different weights to features of different importance levels to improve the classification performance of the model. To further improve accuracy, the FCAN and XGBoost algorithms were fused for emotion recognition. Results obtained from experiments conducted on two datasets, DEAP and DREAMER, demonstrate that the proposed model outperforms existing state-of-the-art models. Specifically, the proposed model achieved an accuracy of 95.26% and 94.05% on the four-class classification task for the DEAP and DREAMER datasets, respectively. Additionally, on the DEAP dataset, our model reduced memory consumption by approximately 92.78% and computing time by 76.70% compared to FCAN–SVM and reduced memory consumption by approximately 70.80% and computing time by 93.47% compared to FCAN–LSTM. On the DREAMER dataset, our model reduced memory consumption by approximately 94.43% and computing time by 75.45% compared to FCAN–SVM and reduced memory consumption by approximately 67.51% and computing time by 81.87% compared to FCAN–LSTM. This indicates that the proposed model significantly reduces computational costs while improving classification accuracy for EEG-based emotion recognition. Furthermore, the proposed model can be generalized to other multi-channel physiological signals for classification and recognition tasks, such as those for motor imagery, fatigue driving detection, and gesture recognition based on physiological signals.

## Figures and Tables

**Table 1:** Detailed parameters of FCN1.

Layer	Layer Setting	Output
Linear1	In_features = 128 Out_feaures = 1024 Activation = ReLU	(128, 1024)
BatchNorm1d	BatchNormalization	(128, 1024)
Dropout	Dropout1D	(128, 1024)
Linear2	In_features = 1024 Out_features = 256 Activation = ReLU	(128, 256)
BatchNorm1d	BatchNormalization	(128, 256)
Dropout	Dropout1D	(128, 256)
Linear3	In_features = 256 Out_features = 128 Activation = ReLU	(128, 128)

**Table 2:** Detailed parameters of FANet.

Layer	Layer Setting	Output
Pool	3, stride = 1; MaxPool	(128, 1, 128)
Conv1D	In_channels = 1; Out_channels = 5; Kernel_size= 63 Stride = 1; Activation = ReLU	(128, 5, 64)
Conv1D	In_channels = 5; Out_channels = 1; Kernel_size= 33 Stride = 1; Activation = ReLU	(128, 1, 32)
Flatten	—	(128, 32)
Linear	In_features = 32; Out_features = 128 Activation = Sigmoid	(32, 128)

**Table 3:** Detailed parameters of FCN2.

Layer	Layer Setting	Output
Linear1	In_features = 256; Out_features = 512; Activation = ReLU	(128, 512)
BatchNorm1d	BatchNormalization	(128, 512)
Dropout	Dropout1D	(128, 512)
Linear2	In_features = 512; Out_features = 1024; Activation = ReLU	(128, 1024)
BatchNorm1d	BatchNormalization	(128, 1024)
Dropout	Dropout1D	(128, 1024)
Linear3	In_features = 1024; Out_features = 256; Activation = ReLU	(128, 256)
BatchNorm1d	BatchNormalization	(128, 256)
Dropout	Dropout1D	(128, 256)
Linear4	In_features = 256; Out_features = 64; Activation = ReLU	(128, 64)
BatchNorm1d	BatchNormalization	(128, 64)
Dropout	Dropout1D	(128, 64)
Linear5	In_features = 64; Out_features = 4; Activation = ReLU	(128, 4)

**Table 4:** Experimental results with different features on DEAP.

Feature	Accuracy/%	Precision/%	Recall/%	F1-Score/%
*DE*	94.40	94.27	94.39	94.33
*PSD*	90.49	90.34	90.37	90.35
$X_{add}$	94.05	94.32	93.67	93.99
$X_{mult}$	95.14	95.13	94.99	95.06
$X_{con}$	95.26	95.15	95.23	95.19

**Table 5:** Experimental results with different features on DREAMER.

Feature	Accuracy/%	Precision/%	Recall/%	F1-Score/%
*DE*	78.39	77.85	73.46	75.59
*PSD*	88.45	88.20	85.90	87.03
$X_{add}$	92.64	92.51	91.65	92.08
$X_{mult}$	92.86	93.53	91.28	92.39
$X_{con}$	94.05	94.87	92.37	93.60

**Table 6:** Comparison experiment results of FANet module on DEAP.

Model	Accuracy/%	Precision/%	Recall/%	F1-Score/%
FCAN–XGBoost	95.26	95.15	95.23	95.19
FCN–XGBoost	94.78	94.73	94.76	94.74
AF	94.69	94.92	94.47	94.69

**Table 7:** Comparison experiment results of FANet module on DREAMER.

Model	Accuracy/%	Precision/%	Recall/%	F1-Score/%
FCAN–XGBoost	94.05	94.87	92.37	93.60
FCN–XGBoost	92.70	93.59	90.84	92.19
AF	92.99	93.82	91.41	92.60

**Table 8:** Comparison experiment results of classifier in different layers.

Dataset	No_FC/%	IFC1/%	IFC2/%	IFC3/%	IFC4/%	IFC5/%
DEAP	92.55	94.34	94.96	94.77	94.64	95.26
DREAMER	88.62	92.44	93.12	92.30	91.67	94.05

**Table 9:** Comparison of results between FCAN–XGBoost and two baseline models on DEAP.

Model	Time (S)	Memory (M)	Accuracy/%	Precision/%	Recall/%	F1-Score/%
FCAN–LSTM	7.35	5.65	94.56	95.04	94.05	94.54
FCAN–SVM	2.06	22.86	91.77	93.85	90.83	92.32
FCAN–XGBoost	0.48	1.65	95.26	95.15	95.23	95.19

**Table 10:** Comparison of results between FCAN–XGBoost and two baseline models on DREAMER.

Model	Time (S)	Memory (M)	Accuracy/%	Precision/%	Recall/%	F1-Score/%
FCAN–LSTM	8.55	5.51	93.24	92.58	92.43	92.50
FCAN–SVM	2.24	32.13	89.22	94.88	83.93	89.07
FCAN–XGBoost	0.55	1.79	94.05	94.87	92.37	93.60

**Table 11:** Performance comparison of state-of-the-art models for four-class classification of valence and arousal on DEAP and DREAMER datasets.

Author	Feature	Classifier	Dataset	Accuracy	Year
Li Z et al. [42]	Standard deviation Difference absolute value Wavelet entropy, Wavelet energy *DE*, *PSD*	MLDW–PSO–SVM	DEAP	76.67%	2020
Zhang J et al. [43]	WPT–DE	RF	DEAP	87.30%	2020
Hou F et al. [44]	Preprocessed Signal matrix (PSM) Symmetric difference matrix (SDM) Symmetric quotient matrix (SQM) DE matrix (DEM)	RFPN–S2D–CNN	DEAP	93.56%	2023
Zhang J et al. [45]	DE	FSA–3D–CNN	DEAP	94.53%	2022
Mehmood R M et al. [16]	Hjorth-feature	WEKA	DEAP	69%	2022
			DREAMER	85%	
Hu Z et al. [46]	Time–frequency map	CNN–BiLSTM–MHSA	DEAP	89.33%	2022
Zhao Y et al. [47]	Spatial-temporal features	3DCNN	DEAP	93.53%	2020
Singh M I et al. [48]	Average event related potentials	SVM	DEAP	75%	2020
	Difference of average ERPs			76.80%	
Zali-Vargahan et al. [50]	Time–frequency feature	SVM	DEAP	88.60%	2023
Liu S et al. [51]	*DE*	GLFANet	DEAP	92.92%	2023
Ours	*DE*, *PSD*	FCAN–SVM	DEAP	91.77%	2023
			DREAMER	89.22%	
		FCAN–LSTM	DEAP	94.56%	
			DREAMER	93.24%	
		FCAN–XGBoost	DEAP	95.26%	
			DREAMER	94.05%	

## Data Availability

The data for this study were obtained from publicly available datasets. The DEAP dataset is available at http://www.eecs.qmul.ac.uk/mmv/datasets/deap/index.html (accessed on 30 November 2022), and the DREAMER dataset is available at https://zenodo.org/record/546113# (accessed on 6 January 2023).

## Abbreviations

The following abbreviations are used in this manuscript:

AI	Artificial Intelligence
EEG	electroencephalogram
*DE*	Differential Entropy
*PSD*	Power Spectral Density
EMG	electromyography
GSR	Galvanic Skin Resistance
ECG	electrocardiogram
SKT	Skin Temperature
VR	Virtual Reality
DEAP	Dataset for Emotion Analysis using Physiological signals
DNN	Deep Neural Networks
CNN	Convolutional Neural Networks
FE	Fuzzy Entropy
MODFA	Multi-Order Detrended Fluctuation Analysis
CAE	Convolutional Autoencoder
SVM	Support Vector Machine
RVM	Relational Vector Machine
GC	Granger Causality
GCN	Graph Convolutional Neural Network
CRNN	Convolutional Recurrent Neural Network
RNN	Recurrent Neural Network
TCNet	Transformer Capsule Network
BiLSTM	Bidirectional Long Short-Term Memory Network
MHSA	Multi-Head Self-Attention
ERPs	Event-Related Potentials
EOG	electrooculogram
SAM	Self-Assessment Manikin
FCN	Fully Connected Neural Network
SAE	Stacked Autoencoder
PCA	Principal Component Analysis
XGBoost	eXtreme Gradient Boosting
GBDT	Gradient Boosting Decision Tree
HVHA	High Valence High Arousal
HVLA	High Valence Low Arousal
LVHA	Low Valence High Arousal
LVLA	Low Valence Low Arousal
RF	Random Forest
LSTM	Long Short-Term Memory
